# Chronic exposure to inhaled vaporized cannabis high in Δ^9^-THC suppresses Adderall-induced brain activity

**DOI:** 10.3389/fphar.2024.1413812

**Published:** 2024-10-10

**Authors:** Jack M. Ognibene, Rajeev I. Desai, Praveen P. Kulkarni, Craig F. Ferris

**Affiliations:** ^1^ Center for Translational NeuroImaging, Northeastern University, Boston, MA, United States; ^2^ Department of Psychiatry, Harvard Medical School, Boston, MA, United States; ^3^ Behavioral Biology Program, Integrative Neurochemistry Laboratory, McLean Hospital, Belmont, MA, United States; ^4^ Departments of Psychology and Pharmaceutical Sciences Northeastern University, Boston, MA, United States

**Keywords:** basal ganglia, functional MRI, awake animal imaging, BOLD, accumbens

## Abstract

**Background:**

There are increasing reports of the misuse of prescription psychostimulants for cognitive enhancement together with recreational cannabis. This raises a concern that chronic use of cannabis high in Δ^9^-THC may alter the sensitivity to amphetamines. In this exploratory study we hypothesized chronic exposure to Δ^9^-THC through vaporized cannabis would diminish the central nervous system (CNS) activity of Adderall.

**Methods:**

To address this issue we exposed male and female mice to inhaled vaporized cannabis (10.3% Δ9-THC) or placebo for 30 min each day for ten consecutive days. After 24 h, mice were imaged fully awake for changes in BOLD signal following an IP injection of Adderall (60 µg) during the scanning session. After a 2-week washout, without any cannabis or placebo exposure, mice were again imaged and challenged with Adderall during the scanning session. The data were registered to a mouse 3D MRI atlas with 134 brain regions providing site-specific increases and decreases in global brain activity.

**Results:**

Mice exposed to cannabis when compared to placebo showed a decrease in brain activation to Adderall. The blunted Adderall response was characterized by a decrease in positive BOLD signal and increase in negative BOLD. The prefrontal cortex, accumbens, ventral pallidum, caudate/putamen, and thalamus were most affected. After a 2-week wash out there were no significant differences between the cannabis and placebo groups when challenged with Adderall.

**Summary:**

This exploratory study shows that short, daily exposures to inhaled cannabis, something equivalent to recreational use, affects the sensitivity to the psychostimulant Adderall. The reduced Adderall effect on brain activity, particularly circuitry associated with dopaminergic signaling raises concerns about escalation in psychostimulant use.

## Introduction

Medical emergencies associated to cannabis, the most widely self-administered psychoactive substance in the United States, has increased dramatically over the last decade. The Drug Abuse Warning Network estimated that in 2021, there were over 785,000 drug-related emergency department visits in the United States in which cannabis use was reported in the medical record (SAMHSA, 2022; SAMHSA, 2011). The prevalence of cannabis use coupled with the medical and legal movement to normalize its use in the United States is especially alarming given that a number of deleterious effects, including higher risk of depression, anxiety, addiction, and psychosis (e.g., (Bechtold et al., 2016; Patton et al., 2002; Han et al., 2019; De Aquino et al., 2018), have been associated with early age of initiation, frequency, and duration of use (Volkow, 2016; Volkow et al., 2014a). The fact that polysubstance use and abuse is the norm rather than the exception in the United States (Kidorf et al., 2018; Lee et al., 2018; Leri et al., 2003; Al-Tayyib et al., 2018; Jones and McCance-Katz, 2019; LaRue et al., 2019), has also raised concerns that a history of cannabis use, and abuse might render individuals more susceptible to consuming other psychoactive substances. Indeed, clinical studies have reported that 75% of cannabis users consume other psychoactive drugs during their lifetime and have a 50% higher cumulative risk in their lifetime of abusing other illicit substances (SAMHSA, 2022; SAMHSA, 2011; Secades-Villa et al., 2015). Of particular concern, is the high incidence of cannabis and psychostimulant use amongst adolescent and college students, including prescription psychostimulants like amphetamine (Adderall) that are typically used as medications for treating Attention-deficit/hyperactivity disorder (ADHD) (Lewis and Martinez, 2023; Asante and Atorkey, 2023).

Despite the expansive clinical and preclinical literature on the neurobiological actions of Δ-9-tetrahydrocannabinol (Δ^9^-THC) – the psychoactive cannabinoid in cannabis (Huestis et al., 2001), remarkably little is known about how a history of cannabis use impacts the neurobiological effects of prescribed psychostimulants like Adderall. As the mesocorticolimbic dopamine (DA) system plays a key role in the abuse-related neurobiological and behavioral effects of psychoactive drugs, including Δ^9^-THC, these neurobiological substrates continue to be a major focus of research for most psychoactive drugs. There is mounting evidence suggesting a complex neurochemical, behavioral, and pharmacological interaction between DA and the cannabinoid (CB) receptor 1 (CB_1_) receptor systems in the brain (Maldonado, 2002; Tanda and Goldberg, 2003). However, next to nothing is known about how a history of chronic exposure to Δ^9^-THC impacts the neurobiological processes involved in subsequent psychomotor stimulant use and abuse. In this regard, in preclinical and clinical studies, an acute dose of Δ^9^-THC has been reported to elevate DA release in the ventral striatum (Bossong et al., 2009; Gardner, 2005; Lupica and Riegel, 2005; Bloomfield et al., 2016). In monkeys, chronic Δ^9^-THC exposure increased DA_1_ and DA_2_ receptor expression in striatal neurons and altered the primate striatal DA_1_ and DA_2_ linked neuron phenotype and signaling (Hasbi et al., 2020). Moreover, some evidence also suggests that heavy, long-term cannabis users manifest reduced DA release following psychostimulant challenge and deficits in DA-related behavioral consequences that conceivably reflect Δ^9^-THC-induced neuroadaptive changes in DA signaling (Volkow, 2016; Volkow et al., 2014a; Bloomfield et al., 2016). However, the link between levels of chronic Δ^9^-THC exposure, dysfunctional DA systems, and Δ^9^-THC-induced functional consequences of prescribed psychostimulant drugs like Adderall remain essentially unknown.

In this exploratory study we used BOLD functional imaging to address the question if chronic exposure to Δ^9^-THC would affect the sensitivity to Adderall. To make the findings relevant to the human experience, mice were exposed to vaporized, cannabis high in Δ^9^-THC once daily for 10 days matching blood levels of drug associated with recreational use. Furthermore, mice were imaged while fully awake when given Adderall during the scanning session. Overall, we found that the activation of the reward-related brain regions with strong dopaminergic innervation, including the prelimbic cortex, ventral pallidum, accumbens and thalamus, was dramatically reduced by Adderall in mice with a history of cannabis inhalation. Interestingly, after discontinuation of inhaled cannabis exposure, the Adderall-induced increases in brain activity in these regions was fully restored.

## Methods and materials

### Animal usage

Approximately 90-day-old female and male C57BL/6J mice (n = 10/sex), weighing between 22–25 g, were procured from Charles River Laboratories (Wilmington, Massachusetts, United States). The mice were subjected to a reverse 12:12 light-dark cycle, with lights turned off at 07:00 h, and were provided with unrestricted access to food and water. All experiments were conducted between 08:00 AM and 06:00 PM to minimize circadian disturbances associated with the light-to-dark transition. The acquisition and care of the mice followed the guidelines outlined in the Guide for the Care and Use of Laboratory Animals (National Institutes of Health Publications No. 85–23, Revised 1985), adhering to the National Institutes of Health and the American Association for Laboratory Animal Science guidelines. The study protocols adhered to the regulations of the Institutional Animal Care and Use Committee at Northeastern University under protocol # 23-0407R, and the research methods complied with the ARRIVE guidelines for reporting *in vivo* experiments in animal research (Kilkenny et al., 2010).

### Drug preparation and administration

#### Cannabis exposure

Ten mice (n = 5 female/n = 5 males) were subjected to cannabis with a high Δ^9^-THC content (10.3% Δ^9^-THC and 0.05% CBD), while another group of ten mice of females and males received placebo cannabis with less than 0.01% Δ^9^-THC and 0.01% CBD. The cannabis was sourced from the National Institute on Drug Abuse (NIH/NIDA, Bethesda, MD) through the Research Triangle Institute (Research Triangle Park, NC). The mice were placed in a 38-L exposure chamber (60 cm × 45 cm × 20 cm), equipped with a vapor inflow tube and several small air outflow holes. To familiarize the subjects with the environment and minimize stress, they were acclimated to the exposure chamber for 2 days before exposure.

A Volcano Vaporizer (Storz and Bickel, Tuttlingen, Germany) was employed to heat cannabis plant material below the point of complete combustion, vaporizing the active ingredient (Δ^9^-THC) and reducing the formation of harmful free radicals like polycyclic aromatic hydrocarbons associated with the combustion of organic plant material. The vaporizer was preheated to approximately 210°C and loaded with 0.450 g of minced cannabis or placebo. Tubing connected the vaporizer to the exposure chamber, and the heating fan ran for a total of 60 s, filling the exposure chamber with vaporized cannabis aerosols. Following 30 min of passive exposure, the mice were removed from the chamber and returned to their cages. This exposure regimen was repeated daily for 10 consecutive days. The amount of minced cannabis used was determined based on prior studies demonstrating that this method produced similar serum Δ^9^-THC concentrations (130–150 ng/mL) to those observed in human users (Farra et al., 2020; Sadaka et al., 2023). Mice were imaged within 24 h after the last exposure. For the administration of the drug during the imaging session from a distance, a polyethylene tube (PE-20) with an approximate length of 30 cm was inserted into the peritoneal cavity. Each mouse was given an injection of 60 µg of *d-l*-amphetamine sulfate (Adderall) (Sigma Aldrich) in a volume of 250 µL of saline vehicle during the scanning session. This dose (2.4 mg/kg) was based on previous preclinical studies that have reported changes in cognitive function and cross-sensitization with other stimulants (Santos et al., 2009; Doremus-Fitzwater and Spear, 2011; Sherrill et al., 2013). Mice were returned to their home cages following imaging and left undisturbed for 2 weeks. After this “washout” period they were imaged again and challenged with an IP injection of Adderall.

#### Awake mouse imaging and acclimation

A comprehensive account of the awake mouse imaging system and the acclimation procedure are detailed elsewhere (Ferris et al., 2014). Mice are acclimated for 1 week prior to imaging. Notably, we utilized a quadrature transmit/receive volume coil with a diameter of 38 mm, offering both high anatomical resolution and a superior signal-to-noise ratio for voxel-based BOLD fMRI. Additionally, the mouse holder’s distinctive design from Ekam Imaging (Boston, MA) ensured complete head stabilization within a cushioned helmet, reducing discomfort associated with conventional ear bars and restraint systems commonly employed for immobilizing the head during awake animal imaging (Ferris, 2022). A visual representation of the mouse setup for awake imaging can be viewed at http://www.youtube.com/watch?v=W5Jup13isqw.

#### Acclimation

One week before the initial imaging session, all mice underwent a familiarization process with the head restraint and the sounds typical of the scanner. Initially, mice were gently secured in the holding system under 1%–2% isoflurane anesthesia. After regaining consciousness, the mice were positioned in a simulated MRI scanner setting for a duration of up to 30 min. This environment resembled a “mock MRI scanner” enclosed in a black box, featuring an audio recording of MRI pulses. The acclimation procedure was repeated daily over four consecutive days to mitigate autonomic nervous system-induced effects during awake animal imaging. Such effects include alterations in heart rate, respiration, corticosteroid levels, and motor movements. The overarching aim was to enhance contrast-to-noise ratios and improve image quality (King et al., 2005). Other research groups have alternatively emphasized more prolonged acclimation periods to minimize stress during awake imaging (Ferris, 2022; Stenroos et al., 2018; Chang et al., 2016).

#### Motion artifact

The awake mouse restraining system combined with acclimation can minimize motion artifact. Supplementary Figure S1 shows the motion artifact as a mean and SE for each of the 250 image acquisitions for all 10 subjects in the two experimental groups–placebo plus Adderall and cannabis plus Adderall. Adderall given to mice exposed to vaporized placebo showed no motion in any orthogonal direction outside 50 um (±) (5.000E-02). The in-plane resolution of a single voxel in this study is ca 187 um^2^. These data show the restraining system, head holder and acclimation procedure effectively minimize any increase in motor activity that may be caused by Adderall. When mice are exposed to vaporized cannabis daily for 10 days and then withheld for 24 h–there is increased motion in X and Y due to slight rotation of the head, hence the mirror image of the red and black lines. These spikes between 60–70, 91–101 and plateau starting at 141 reach 100 um (±) (1.000E-01) are just over ½ of a voxel dimension. These changes are judged to be acceptable as most correction algorithms can adjust for motion artifact when movement is below the size of a voxel. The difference between the two experimental conditions is noteworthy because it may reflect withdrawal from chronic cannabis exposure, something not reported in the preclinical imaging literature. We observed a similar increase in motion artifact in another study following a 24 h hiatus from chronic oxycodone exposure (Iriah et al., 2019). In that case the motion exceed the dimensions of a voxel and the data were unusable. In the absence of additional data, this is purely speculative and will require further research.

#### BOLD image acquisition and pulse sequence

Experiments were conducted using a Bruker Biospec 7.0T/20-cm USR horizontal magnet (Bruker; Billerica, MA) and a 2 T/m magnetic field gradient insert (ID = 12 cm) capable of a 120-µsec rise time (Sadaka et al., 2021). At the beginning of each imaging session, a high-resolution anatomical data set was collected using the rapid acquisition relaxation enhancement (RARE) pulse sequence (RARE factor 8); (18 slices; 0.75 mm; field of view (FOV) 1.8 cm^2^; data matrix 128 × 128; repetition time (TR) 2.1 s; echo time (TE) 12.4 ms; Effect TE 48 ms; number of excitations (NEX) 6; 6.5 min acquisition time). Functional images were acquired using a multi-slice Half Fourier Acquisition Single Shot Turbo Spin Echo (HASTE) pulse sequence (RARE factor 53); (18 slices; 0.75 mm; FOV 1.8 cm; data matrix 96 × 96; TR 6 s; TE 4 ms; Effective TE 24 ms; 25 min acquisition time; in-plane resolution 187.5 µm^2^). This spatial resolution is enough to delineate the bilateral habenula (ca 4-5 voxels for each side) but not between lateral and medial habenula. The lateral habenula is involved in coordinating a response to aversive stimuli by affecting activity in the ventral tegmental area and substantia nigra (Stamatakis and Stuber, 2012). The medial habenula has no such role. We hold the habenula up as an example of the spatial limitations of preclinical fMRI. Each functional imaging session consisted of uninterrupted data acquisitions (whole brain scans) of 250 scan repetitions or acquisitions for a total elapsed time of 25 min. The control window included the first 50 scan acquisitions (18 slices acquired in each), covering a 5 min baseline. Following the control window, an I.P. injection of Adderall was given followed by another 200 acquisitions over a 20 min period.

#### Imaging data analysis

The impact of Adderall on brain activity was measured by quantifying positive and negative percent changes in the BOLD signal compared to the baseline. Initial analyses of signal changes in individual subjects involved comparing image acquisitions 125–225 to the baseline 1–45. The statistical significance of these changes was evaluated for each voxel (approximately 15,000 per subject in their original reference system) using independent Student t-tests, employing a 1% threshold to account for normal fluctuations of the BOLD signal in the awake rodent brain. The steps taken to control for multiple t-tests and false-positive detections have discussed in detail in previous studies (Sadaka et al., 2023; Sathe et al., 2023). Voxel-based percent changes in the BOLD signal, generated for individual subjects, were aggregated across subjects within the same group to construct representative functional maps. Image registration, alignment, and percentage change in BOLD signal for each voxel using a 3D Mouse Brain Atlas^©^ with 134 segmented and annotated brain regions (Ekam Solutions; Boston, MA) has been described in previous studies (Sadaka et al., 2023; Sathe et al., 2023). The Kruskal-Wallis test statistic was employed to compare the number of activated voxels in each of the 134 regions between the placebo and cannabis groups.

## Results

**FIGURE 1:**
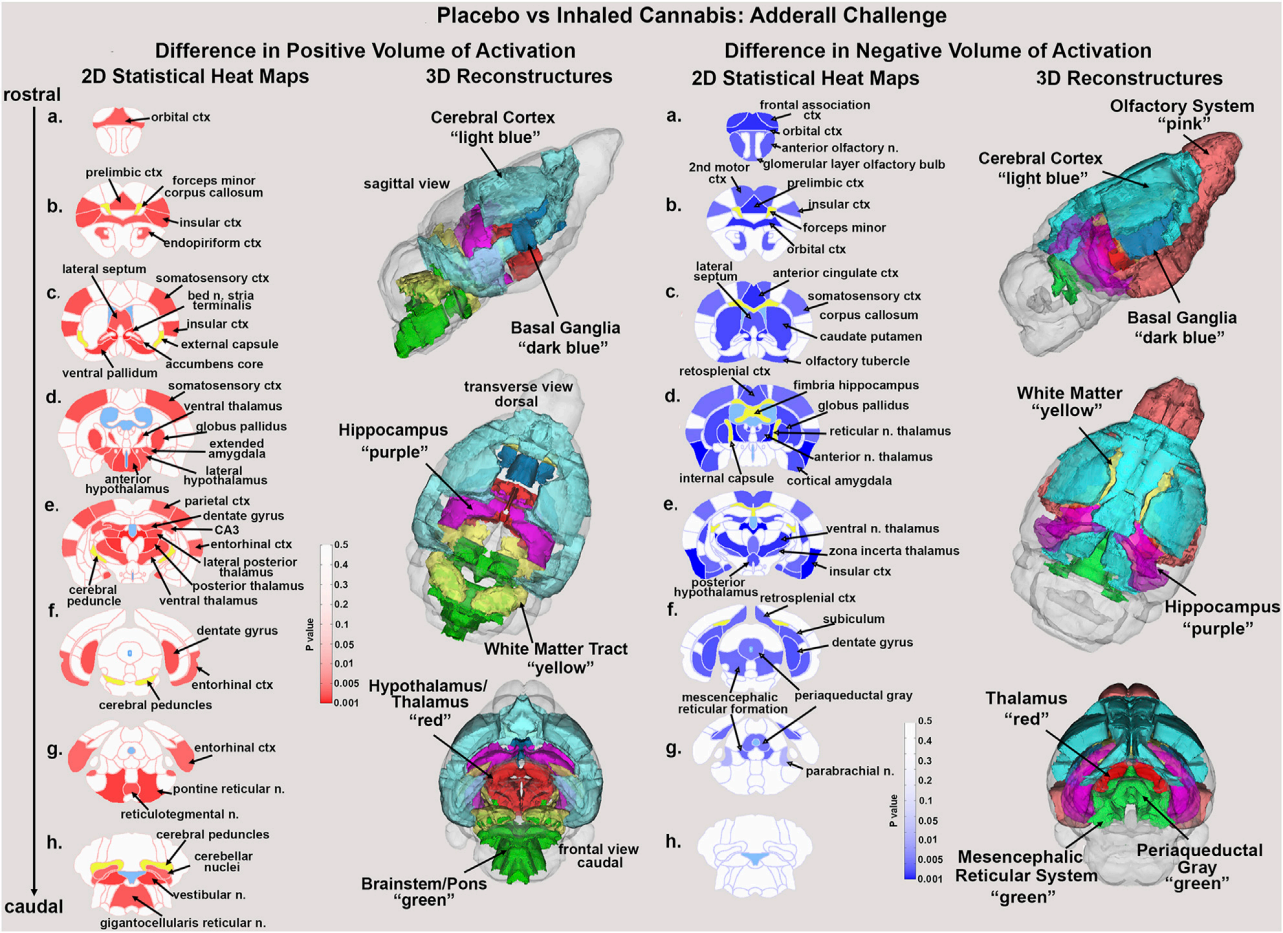
Statistical heat maps. Shown are 2D statistical heat maps for positive BOLD volume of activation (red) and negative BOLD volume of activation (blue). The areas shown were significantly different between mice exposed to placebo or cannabis and challenged with Adderall. The 3D color coded reconsrtuctions are a summary of the signficanlty affected areas from the 2D maps. ctx: cortex, n. nucleus.

Tables reporting the positive and negative volumes of activation, i.e., number of voxels activated for all 134 brain areas for placebo and cannabis are provided in Supplementary Tables S1A, B. When the data from both groups are collapsed and separated in males and females there are no significant differences between sexes (Supplementary Material S1). There was a significant decrease in positive volume of activation following Adderall injection in mice with a history of cannabis exposure as shown in Table 1. This Adderall response affected 32/134 brain regions. These areas are ranked in order of their significance. Reported is the average (Ave) and standard error (SE) for placebo and cannabis groups together with probability values and the omega square (ω Sq) for effect size. The critical value was set at *p* < 0.05. A false discovery rate (FDR) for multi-comparisons gave a significant level of *p* = 0.046. The thalamus showed reduced activity with Adderall e.g., anterior pretectal, posterior, ventral, lateral posterior and central medial nuclei. Also affected were brain areas associated with the ascending reticular activating system and brain arousal e.g., medullary reticular n., pontine reticular n., parabrachial n. and gigantocellularis reticularis. It should also be noted that the accumbens and ventral pallidum, brain areas with dopaminergic efferent connections from the ventral tegmental area showed decreased activation with Adderall. The location of these areas and others from Table 1 are shown in 2D statistical heat maps in Figure 1.

**TABLE 1:** Positive BOLD volume of activation: Adderall challenge (number of positive voxels).

Brain area	Placebo			Cannabis		P-val	Ω Sq
	Ave	SE		Ave	SE		
medullary reticular ventral n.	**31**	**6.6**	**>**	**6**	**3.8**	**0.009**	**0.331**
anterior pretectal thalamic n.	**18**	**2.6**	**>**	**7**	**2.0**	**0.014**	**0.285**
bed n. stria terminalis	**41**	**5.1**	**>**	**20**	**5.7**	**0.016**	**0.272**
posterior thalamic n.	**25**	**3.1**	**>**	**9**	**3.6**	**0.019**	**0.254**
pontine reticular n. oral	**110**	**14.7**	**>**	**45**	**18.1**	**0.022**	**0.241**
ventral pallidum	**56**	**7.5**	**>**	**24**	**9.3**	**0.023**	**0.234**
vestibular n.	**63**	**11.8**	**>**	**26**	**10.6**	**0.024**	**0.231**
anterior hypothalamic n.	**65**	**8.5**	**>**	**38**	**7.7**	**0.027**	**0.218**
lateral posterior thalamic n.	**21**	**2.7**	**>**	**7**	**3.3**	**0.029**	**0.017**
globus pallidus	**73**	**8.7**	**>**	**29**	**11.7**	**0.030**	**0.209**
insular rostral ctx	**159**	**19.8**	**>**	**88**	**23.1**	**0.030**	**0.208**
parabrachial n.	**16**	**2.5**	**>**	**7**	**3.4**	**0.031**	**0.207**
prelimbic ctx	**43**	**5.7**	**>**	**17**	**7.0**	**0.033**	**0.200**
ventral thalamic n.	**151**	**18.1**	**>**	**65**	**24.1**	**0.033**	**0.199**
dentate gyrus	**186**	**21.6**	**>**	**81**	**29.4**	**0.034**	**0.198**
primary somatosensory ctx	**579**	**71.9**	**>**	**299**	**91.4**	**0.034**	**0.198**
forceps minor corpus callosum	**7**	**1.4**	**>**	**2**	**0.9**	**0.035**	**0.194**
central medial thalamic n.	**14**	**2.1**	**>**	**6**	**2.4**	**0.035**	**0.194**
reticulotegmental n.	**25**	**3.9**	**>**	**9**	**4.4**	**0.035**	**0.193**
gigantocelllaris reticular n.	**123**	**22.9**	**>**	**53**	**22.2**	**0.037**	**0.190**
lateral septal n.	**67**	**8.7**	**>**	**34**	**10.1**	**0.037**	**0.189**
accumbens core	**36**	**5.0**	**>**	**14**	**6.0**	**0.039**	**0.183**
extended amygdala	**22**	**3.4**	**>**	**10**	**3.8**	**0.039**	**0.183**
orbital ctx	**157**	**21.3**	**>**	**65**	**26.7**	**0.041**	**0.179**
lateral rostral hypothalamic n.	**84**	**11.1**	**>**	**36**	**13.7**	**0.041**	**0.179**
CA3	**83**	**11.3**	**>**	**40**	**12.0**	**0.041**	**0.178**
dorsal hippocampal commissure	**8**	**1.0**	**>**	**4**	**1.3**	**0.043**	**0.175**
endopiriform n.	**23**	**2.9**	**>**	**10**	**4.0**	**0.046**	**0.167**
cerebellar nuclear n.	**35**	**6.0**	**>**	**14**	**6.8**	**0.049**	**0.163**
parietal ctx	**7**	**1.5**	**>**	**3**	**1.4**	**0.049**	**0.162**
cerebral peduncle	**136**	**19.7**	**>**	**61**	**24.0**	**0.050**	**0.160**
entorhinal ctx	**425**	**53.6**	**>**	**234**	**61.6**	**0.050**	**0.160**

Shown in Table 2 are the changes in negative volume of activation. There were 42/134 brain areas that showed a significant increase in negative BOLD volume of activation (FDR = 0.058). Several of these areas matched those in Table 1 e.g., prelimbic ctx, bed n. stria terminalis, dentate gyrus. Again the thalamus was well represented in addition to the prefrontal ctx, e.g., orbital, anterior cingulate, frontal association, prelimbic and 2^nd^ motor cortices. The olfactory system also showed an increase in negative BOLD volume of activation e.g., glomerular layer of the olfactory bulb, anterior olfactory n., piriform cortices, and cortical amygdala.

**TABLE 2:** Negative volume of activation: Adderall challenge (number of negative voxels).

Brain area	Placebo			Cannabis		P-val	Ω Sq
	Ave	SE		Ave	SE		
reticular thalamic n.	**1**	**0.8**	**<**	**8**	**2.9**	**0.006**	**0.342**
rostral piriform ctx	**6**	**5.6**	**<**	**51**	**21.6**	**0.006**	**0.330**
internal capsule	**2**	**1.9**	**<**	**15**	**4.5**	**0.008**	**0.316**
lemniscal n.	**0**	**0.0**	**<**	**7**	**3.4**	**0.008**	**0.281**
anterior pretectal thalamic n.	**1**	**0.6**	**<**	**6**	**1.6**	**0.009**	**0.318**
orbital ctx	**3**	**2.4**	**<**	**51**	**21.4**	**0.010**	**0.296**
bed n. stria terminalis	**2**	**2.3**	**<**	**9**	**3.6**	**0.011**	**0.297**
prelimbic ctx	**1**	**1.3**	**<**	**13**	**5.0**	**0.012**	**0.273**
ventral thalamic n.	**5**	**4.7**	**<**	**44**	**16.3**	**0.012**	**0.273**
anterior cingulate n.	**3**	**2.5**	**<**	**24**	**10.7**	**0.013**	**0.266**
globus pallidus	**2**	**1.3**	**<**	**24**	**9.2**	**0.013**	**0.275**
frontal association ctx	**4**	**3.8**	**<**	**31**	**10.5**	**0.016**	**0.259**
caudate putamen	**19**	**15.7**	**<**	**177**	**66.2**	**0.020**	**0.235**
cortical amygdaloid n.	**6**	**4.2**	**<**	**24**	**7.5**	**0.022**	**0.218**
anterior amygdaloid n.	**0**	**0.0**	**<**	**3**	**1.5**	**0.022**	**0.172**
dentate gyrus	**4**	**3.8**	**<**	**42**	**18.7**	**0.022**	**0.231**
dorsal hippocampal commissure	**0**	**0.0**	**<**	**3**	**1.4**	**0.022**	**0.188**
forceps minor corpus callosum	**0**	**0.0**	**<**	**3**	**1.3**	**0.022**	**0.194**
olfactory tubercles	**0**	**0.1**	**<**	**6**	**4.3**	**0.023**	**0.201**
medial dorsal thalamic n.	**1**	**1.2**	**<**	**5**	**1.5**	**0.024**	**0.206**
caudal piriform ctx	**8**	**6.2**	**<**	**37**	**10.7**	**0.025**	**0.209**
medullary reticular ventral n.	**4**	**3.6**	**<**	**12**	**4.9**	**0.027**	**0.216**
retrosplenial rostral ctx	**9**	**7.1**	**<**	**39**	**15.7**	**0.027**	**0.203**
anterior thalamic n.	**3**	**2.4**	**<**	**9**	**2.9**	**0.027**	**0.201**
corpus callosum	**7**	**4.9**	**<**	**38**	**15.6**	**0.027**	**0.204**
lateral septal n.	**2**	**1.6**	**<**	**18**	**7.1**	**0.027**	**0.208**
subiculum	**3**	**2.6**	**<**	**62**	**28.3**	**0.029**	**0.180**
zona incerta	**0**	**0.2**	**<**	**9**	**3.9**	**0.029**	**0.176**
anterior olfactory n.	**8**	**7.2**	**<**	**49**	**21.2**	**0.031**	**0.193**
secondary motor ctx	**6**	**5.4**	**<**	**54**	**21.4**	**0.037**	**0.172**
medial geniculate	**0**	**0.2**	**<**	**11**	**5.4**	**0.038**	**0.159**
mesencephalic reticular formation	**3**	**3.0**	**<**	**62**	**29.4**	**0.038**	**0.156**
retrosplenial caudal ctx	**9**	**9.3**	**<**	**46**	**20.1**	**0.038**	**0.161**
basal amygdaloid n.	**2**	**1.6**	**<**	**15**	**4.5**	**0.038**	**0.171**
entorhinal ctx	**23**	**14.9**	**<**	**111**	**41.1**	**0.038**	**0.178**
fimbria hippocampus	**6**	**3.1**	**<**	**17**	**4.8**	**0.038**	**0.173**
flocculus cerebellum	**11**	**5.9**	**<**	**39**	**12.5**	**0.038**	**0.173**
primary somatosensory ctx	**17**	**11.1**	**<**	**180**	**73.5**	**0.042**	**0.174**
insular caudal ctx	**2**	**1.4**	**<**	**15**	**6.2**	**0.042**	**0.162**
periaqueductal gray	**7**	**6.0**	**<**	**29**	**13.3**	**0.047**	**0.157**
glomerular layer	**11**	**6.3**	**<**	**70**	**27.2**	**0.047**	**0.158**
parabrachial n.	**1**	**1.2**	**<**	**6**	**2.2**	**0.048**	**0.147**

Shown in Figure 1 are the anatomical localization of the areas listed in Tables 1, 2. The coronal sections are statistical heat maps labeled as sections a.- h. and arranged from rostral (top) to caudal (bottom). Light blue denotes the location of cerebroventricles and yellow denotes white matter tracts. The left side (red highlights) shows brain areas that had a decrease in positive BOLD signal with Adderall treatment while the right side shows areas that had an increase in negative BOLD with Adderall treatment. Together, this pattern of BOLD signal change would indicate chronic exposure to cannabis reduces the stimulant activity of Adderall. Sections (a. and b. left and right) highlight changes in the prefrontal cortex, e.g., 2^nd^ motor, orbital, frontal association, prelimbic and insular cortices and the forceps minor, white matter projections to the prefrontal ctx. Section (c. left and right) highlights the accumbens core, ventral pallidum and caudate, all areas with dopaminergic efferent connections from the ventral tegmental area. Sections (d. and e. left and right) show the many thalamic areas affected by Adderall treatment. Sections (e. and f. left and right) highlights the hippocampus e.g., CA3, subiculum and dentate gyrus. Sections (g. and h.) show pons and brainstem. The 3D color coded reconstructions summarize the data from Tables 1, 2.

**FIGURE 2:**
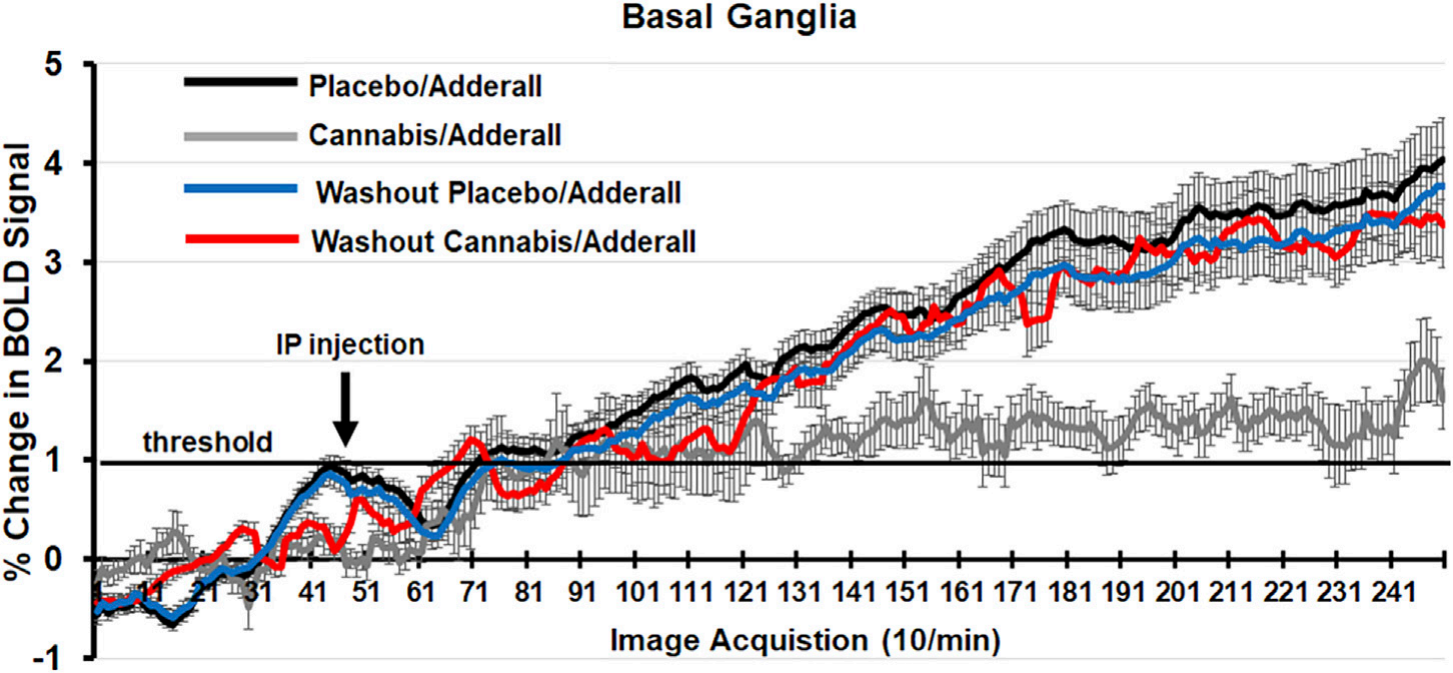
Change in BOLD signal over time. Shown are time series of percentage change in BOLD signal over the 25 min scanning session (250 image acquisitions) for mice exposed to chronic placebo and challenged with Adderall before (black line) and after (blue line) the 2-week washout and mice exposed to chronic cannabis and challenged with Adderall before (gray line) and after (red line) washout. These time series were generated by averaging the BOLD signal at each image acquisition from the accumbens, caudate/putamen and ventral pallidum, areas comprising the basal ganglia. Adderall was injected (arrow) at 5 min (50 acquisitions). The 1% threshold is highlighted by the black line to account for the normal fluctuations in BOLD signal in the awake mouse brain.

Shown in Figure 2 are time series of percentage change in BOLD signal over the 25 min scanning session for mice exposed to chronic placebo and challenged with Adderall before (black line) and after (blue line) the 2-week washout and mice exposed to chronic cannabis and challenged with Adderall before (gray line) and after (red line) washout. These time series were generated by averaging the BOLD signal at each image acquisition from the accumbens, caudate/putamen and ventral pallidum, areas comprising the basal ganglia highlighted in Tables 1, 2. Each mouse from each experimental condition (n = 9 for chronic cannabis; n = 10 for chronic placebo) was represented in the time series. Adderall was injected (arrow) at 5 min (50 acquisitions). The 1% threshold is highlighted by the black line to account for the normal fluctuations in BOLD signal in the awake mouse brain. A two-way ANOVA showed a significant interaction between time and experimental condition for placebo/Adderall vs. cannabis/Adderall [F (_249, 18,177)_ = 8.979, *p* < 0.0001]. There were no significant differences between experimental conditions after washout (See Table 3).

**TABLE 3:** Two-week washout: Positive and negative BOLD volume of activation with Adderall challenge. (Number of positive and negative voxels).

Positive volume of activation							
Brain n.	Placebo			Cannabis		P-val	Ω Sq
	Ave	SE		Ave	SE		
locus coeruleus	**1**	**0.4**	>	**0**	**0.0**	**0.001**	**1.000**
lateral lemniscus	**13**	**2.3**	<	**18**	**3.2**	**0.036**	**0.071**
frontal association ctx	**79**	**13.3**	>	**59**	**12.6**	**0.138**	**0.063**

Negative volume of activation							
Brain n.	Placebo			Cannabis		P-val	Ω Sq
	Ave	SE		Ave	SE		
anterior amygdaloid n.	**0**	**0.0**	**<**	**1**	**1.0**	**0.109**	**−0.055**
accumbens core	**0**	**0.0**	**<**	**1**	**1.1**	**0.109**	**−0.057**

## Discussion

The global use of cannabis and amphetamines is an international public health problem (UNODC, 2016). Amongst adolescent and college students there is a high incidence of cannabis and amphetamine use (Lewis and Martinez, 2023; Asante and Atorkey, 2023). Considering the combined use of stimulants to enhance cognitive performance with the recreational use of cannabis, we sought to evaluate how chronic cannabis use will impact Adderall-induced changes on brain activity. Remarkably, we found that the effects of Adderall on BOLD functional activity in reward-related brain regions (e.g., prelimbic cortex, ventral pallidum, accumbens, and thalamus) was completely abolished in both male and female mice exposed to cannabis compared to placebo. However, after a 2-week discontinuation of Δ^9^-THC, both the Δ^9^-THC- and placebo-exposed mice showed robust and comparable Adderall-induced functional brain activity changes in these reward-related regions. Given the importance of DA in the effects of psychomotor stimulants, our data suggest that Δ^9^-THC use likely triggers neurobiological adaptations that switch the DA reward-related brain regions off, rendering them unresponsive to Adderall, and that discontinuation of Δ^9^-THC use “reactivates” these brain regions leading to a restoration of Adderall’s responsiveness.

There have been numerous BOLD imaging studies in rats following changes in brain activity with amphetamine or methylphenidate (Ritalin) challenge (Easton et al., 2009; Easton et al., 2007; Preece et al., 2007; Chen et al., 1997; Dixon et al., 2005). While all these studies have been conducted under anesthesia, they report activation in the accumbens, caudate/putamen, thalamus and prefrontal cortex as demonstrated in this study on awake mice. Studies on human volunteers show amphetamine enhances activity in the prefrontal cortex and striatum through a DA mechanism (Slifstein et al., 2015; O’Daly et al., 2014). Psychostimulants enhance cognition and attention by activating the prefrontal cortex and the extended frontostriatal circuit (Spencer et al., 2015). In an earlier study, we treated juvenile rats with methylphenidate or amphetamine through the peripubertal period and discovered that the normal functional connections between the striatum, sensorimotor cortices and prefrontal cortex were reduced (Demaree et al., 2021). To the best of our knowledge, this is the first MRI study of chronic Δ^9^-THC exposure and discontinuation on mice, awake or anesthetized, challenged with amphetamine.

It is noteworthy that all substances of abuse impact the functioning of brain regions with strong dopaminergic innervation, causing neuroadaptive alterations associated with drug reinforcement (Koob and Volkow, 2016). Prolonged cannabis use heightens the susceptibility to substance abuse and dependence (Volkow et al., 2014b; Ramesh et al., 2011). Cannabis has the shortest duration from first use to dependence, and earlier onset of use presents an elevated risk for developing dependence (Behrendt et al., 2009). There is ample evidence that the effects of chronic exposure to Δ^9^-THC on tolerance and dependence is due to cannabinoid (CB) receptor 1 (CB_1_) downregulation/desensitization in specific regions of the brain (Panagis et al., 2008; Singh et al., 2011; McMahon, 2011). Studies in humans have shown that frequent exposure to CB_1_ agonists such as Δ^9^-THC leads to physical dependence and withdrawal (Panagis et al., 2008; Singh et al., 2011; McMahon, 2011; Haney et al., 1999; Budney et al., 2004; Beardsley et al., 1986; Desai et al., 2013; Stewart and McMahon, 2010). Continuous exposure to Δ^9^-THC in mice and rat’s results in physical dependence as well (Manwell et al., 2014; Wilson et al., 2006; Bruijnzeel et al., 2016). Research by Freels and colleagues demonstrated that vaporized cannabis extracts possess reinforcing properties, supporting conditioned drug-seeking behavior in rats (Freels et al., 2020). In previous experiments, we exposed both young adult and elderly mice to daily inhaled vaporized cannabis for two to three consecutive weeks. Utilizing voxel-based morphometry and diffusion-weighted imaging, we observed structural changes in the midbrain dopaminergic system (Sadaka et al., 2023; Taylor et al., 2023). The responsiveness of these brain areas to Δ^9^-THC aligns with findings in preclinical literature (Kolb et al., 2006; Madularu et al., 2017).

In the present study, using BOLD functional imaging, we find the enhanced change in signal to Adderall is abolished in mice exposed to chronic inhaled cannabis. To be clear, when comparing the Adderall response in mice with a history of placebo and history of cannabis the positive volume of activation–the increase in number of positive BOLD voxels–decreased indicating that Adderall is less effective in stimulating brain activity. This decrease in brain activity to Adderall in mice with a history of cannabis exposure is complimented by an increase in negative BOLD volume of activation as these voxel numbers rise. In this case, Adderall is presumably reducing brain activity and decreasing blood flow. The accumbens, ventral pallidum and caudate/putamen, targets of DA efferents from the midbrain DA neurons, show reduced sensitivity to Adderall. This blunted response to Adderall also included the prefrontal cortex and thalamus. As we noted earlier, there is no established link between cannabis and amphetamine that could explain these results. The most plausible explanation is disruption in DA signaling. Indeed, the heightened motion artifact in the cannabis group as compared to placebo would be evidence to that effect. What was most interesting is the presumed flexibility, in that, following a 2-week discontinuation of cannabis exposure, the Adderall-induced changes in brain activity in the reward-related regions was fully restored, presumably due to a restoration of activity within the DA system. There is precedent for this flexibility in the DA system as reported in old mice exposed to vaporized cannabis for 4 weeks. Voxel based morphometry showed the areas comprising the DA system were smaller as compared to placebo. However, after a 2-week washout these measures of brain volume were reversed–now larger than placebo (Sadaka et al., 2023).

### Data interpretation, limitations, considerations, and future studies

We recognize that the findings presented in this study raises several important and fundamental questions regarding chronic Δ^9^-THC exposure and psychomotor stimulant use and abuse. For example, Adderall is believed to enhance cognitive function in the prefrontal cortex (PFC) by inhibiting the reuptake of monoamines, including DA, serotonin, and norepinephrine (Easton et al., 2009; Easton et al., 2007), thereby increasing neurotransmission of these chemical signals (Preece et al., 2007; Chen et al., 1997). It is worth noting, that these initial neuroimaging studies were designed to document changes in brain activity in reward-related brain regions rather than changes in monoamine levels, which would require another level of analysis (e.g., *in vivo* microdialysis) that was beyond the scope of this work. Although speculative in the absence of additional neurochemical data, the present findings suggest that short-term monoamine adaptations, especially in DA activity, are likely to occur in the reward-related brain regions. Also, the relationship between structural changes in the DA-related system due to chronic Δ^9^-THC exposure, subsequent Adderall use, and cognitive function remains unclear. While the sensitivity to Adderall was significantly affected by chronic cannabis exposure, here we did not evaluate disruptions in cognitive performance and therefore it would be difficult for us to speculate how chronic Δ^9^-THC and/or Adderall use impacts cognitive processes.

The observation that Adderall’s responsiveness on brain activity was fully restored following cessation of Δ^9^-THC was surprising and somewhat remarkable. At present, it is unclear whether this reflects neuroplasticity *per se*, given that chronic Δ^9^-THC leads to differential tolerance or cross-tolerance among CB1-agonists across various behavioral, pharmacological, and physiologic endpoints that may reflect regional differences in CB1 receptor downregulation/desensitization in the CNS and/or pharmacological efficacy. Studies in humans and laboratory animals have also shown that frequent exposure to CB1 agonists such as Δ^9^-THC leads to physical dependence and withdrawal. Although mounting evidence suggests that complex neurochemical, behavioral, and pharmacological interactions exists between DA–CB1 receptor systems in the brain, few studies have directly investigated the interplay between these systems, and next to nothing is known about how a history of heavy chronic exposure to Δ^9^-THC impacts subsequent stimulant neurobiology in both sexes. Of interest, one interpretation of our data may be that neuroadaptations in dopaminergic activity may offer the opportunity for pharmacological interventions for substance use disorders; however, such an interpretation would be speculative as these experiments have not yet been conducted. An important consideration regarding our observed data is whether greater levels of cannabis exposure cause more long-term neuroadaptive changes in DA system that cannot be restored. Additional studies are needed to fully address these issues. To that end, postmortem histology studies would have helped to identify these changes at a molecular level within the monoamine systems in reward-related brain areas.

What would the Adderall effect have been if it followed a single exposure to vaporized cannabis? It would have been interesting to see how cannabis and Adderall interacted if they had been given together on a daily schedule for a prolonged period. The presentation of Adderall 24 h after a single exposure to inhaled cannabis would not be expected to interfere with the stimulant activities of this drug. The developmental studies we have reported in the literature giving cannabis for 2–3 weeks alters the DA system. There is no evidence this happens with a single exposure of cannabis.

With the legalization of cannabis in much of the US, its recreational use can be a daily routine among many individuals. Moreover, 75% of cannabis users consume other drugs of abuse and have a 50% higher cumulative risk in their lifetime of abusing other addictive substances. Indeed, it is common practice for individuals that use cannabis in high school, college, professional schools, and on the job to also use psychomotor stimulants [e.g., Adderall; amphetamines] either recreationally or therapeutically for treating ADHD and narcolepsy. Although both cannabis and psychomotor stimulant polysubstance misuse is the norm rather than the exception, remarkably little is known about how exposure to Δ^9^-THC with intermittent use of Adderall (i.e., recreational scenario), vs. chronic exposure of Δ^9^-THC together with Adderall (i.e., therapeutic scenario) will, respectively, produce short- and long-lasting adaptations in DA-related neuroimaging and neurochemical signatures to impact cognition. Additional studies are needed to meaningfully address this issue.

Finally, blood concentrations of Δ^9^-THC were not specifically gauged but were presumed to be similar to those reported in our prior studies using male and female mice who underwent inhalation in a chamber using the vaporization technique with a 10.3% Δ^9^-THC cannabis mass (Sadaka et al., 2023). It is possible that the daily exposure to cannabis may have altered the pharmacokinetics of Δ^9^-THC, such that a 24 h discontinuation may not have been sufficient to eliminate Δ^9^-THC from brain and blood. Also, the Adderall effect would have the confound of Δ^9^-THC on-board. Moreover, we did not measure plasma levels of amphetamine and show the equivalence to human usage as we did for Δ^9^-THC.

## Summary

This study provides clear evidence that the single exposure to cannabis each day for several days reduces the effect of Adderall on the prefrontal cortex, ventral striatal/caudate putamen, sensorimotor circuit. The use of vaporized cannabis that presumably produces blood levels of Δ^9^-THC found in humans following smoked cannabis for recreational use reflects the human experience. These data have important clinical implications and inform the public regarding the risk of combining the recreational use of cannabis with psychostimulants to enhance cognitive performance or in the treatment of ADHD.

## Data Availability

The original contributions presented in the study are included in the article/Supplementary Material, further inquiries can be directed to the corresponding author.

## References

[B1] Al-Tayyib A. Riggs P. Mikulich-Gilbertson S. Hopfer C. (2018). Prevalence of nonmedical use of prescription opioids and association with Co-occurring substance use disorders among adolescents in substance use treatment. J. Adolesc. Health 62 (2), 241–244. 10.1016/j.jadohealth.2017.09.018 29174697 PMC5803361

[B2] Asante K. O. Atorkey P. (2023). Cannabis and amphetamine use among school-going adolescents in sub-Saharan Africa: a multi-country analysis of prevalence and associated factors. BMC psychiatry 23 (1), 778. 10.1186/s12888-023-05283-w 37875858 PMC10599041

[B3] Beardsley P. M. Balster R. L. Harris L. S. (1986). Dependence on tetrahydrocannabinol in rhesus monkeys. J. Pharmacol. Exp. Ther. 239 (2), 311–319. 3021952

[B4] Bechtold J. Hipwell A. Lewis D. A. Loeber R. Pardini D. (2016). Concurrent and sustained cumulative effects of adolescent marijuana use on subclinical psychotic symptoms. Am. J. Psychiatry 173 (8), 781–789. 10.1176/appi.ajp.2016.15070878 27138587 PMC5390686

[B5] Behrendt S. Wittchen H. U. Hofler M. Lieb R. Beesdo K. (2009). Transitions from first substance use to substance use disorders in adolescence: is early onset associated with a rapid escalation? Drug alcohol dependence 99 (1-3), 68–78. 10.1016/j.drugalcdep.2008.06.014 18768267

[B6] Bloomfield M. A. Ashok A. H. Volkow N. D. Howes O. D. (2016). The effects of Δ9-tetrahydrocannabinol on the dopamine system. Nature 539 (7629), 369–377. 10.1038/nature20153 27853201 PMC5123717

[B7] Bossong M. G. van Berckel B. N. Boellaard R. Zuurman L. Schuit R. C. Windhorst A. D. (2009). Delta 9-tetrahydrocannabinol induces dopamine release in the human striatum. Neuropsychopharmacology 34 (3), 759–766. 10.1038/npp.2008.138 18754005

[B8] Bruijnzeel A. W. Qi X. Guzhva L. V. Wall S. Deng J. V. Gold M. S. (2016). Behavioral characterization of the effects of cannabis smoke and anandamide in rats. PLoS One 11 (4), e0153327. 10.1371/journal.pone.0153327 27065006 PMC4827836

[B9] Budney A. J. Hughes J. R. Moore B. A. Vandrey R. (2004). Review of the validity and significance of cannabis withdrawal syndrome. Am. J. Psychiatry 161 (11), 1967–1977. 10.1176/appi.ajp.161.11.1967 15514394

[B10] Chang P. C. Procissi D. Bao Q. Centeno M. V. Baria A. Apkarian A. V. (2016). Novel method for functional brain imaging in awake minimally restrained rats. J. Neurophysiol. 116 (1), 61–80. 10.1152/jn.01078.2015 27052584 PMC4961750

[B11] Chen Y. C. Galpern W. R. Brownell A. L. Matthews R. T. Bogdanov M. Isacson O. (1997). Detection of dopaminergic neurotransmitter activity using pharmacologic MRI: correlation with PET, microdialysis, and behavioral data. Magn. Reson Med. 38 (3), 389–398. 10.1002/mrm.1910380306 9339439

[B12] De Aquino J. P. Sherif M. Radhakrishnan R. Cahill J. D. Ranganathan M. D'Souza D. C. (2018). The psychiatric consequences of cannabinoids. Clin. Ther. 40 (9), 1448–1456. 10.1016/j.clinthera.2018.03.013 29678279

[B13] Demaree J. L. Ortiz R. J. Cai X. Aggarwal D. Senthilkumar I. Lawson C. (2021). Exposure to methylphenidate during peri-adolescence decouples the prefrontal cortex: a multimodal MRI study. Am. J. Transl. Res. 13 (7), 8480–8495. 34377346 PMC8340152

[B14] Desai R. I. Thakur G. A. Vemuri V. K. Bajaj S. Makriyannis A. Bergman J. (2013). Analysis of tolerance and behavioral/physical dependence during chronic CB1 agonist treatment: effects of CB1 agonists, antagonists, and noncannabinoid drugs. J. Pharmacol. Exp. Ther. 344 (2), 319–328. 10.1124/jpet.112.198374 23197773 PMC3558821

[B15] Dixon A. L. Prior M. Morris P. M. Shah Y. B. Joseph M. H. Young A. M. (2005). Dopamine antagonist modulation of amphetamine response as detected using pharmacological MRI. Neuropharmacology 48 (2), 236–245. 10.1016/j.neuropharm.2004.10.006 15695162

[B16] Doremus-Fitzwater T. L. Spear L. P. (2011). Amphetamine-induced incentive sensitization of sign-tracking behavior in adolescent and adult female rats. Behav. Neurosci. 125 (4), 661–667. 10.1037/a0023763 21534648 PMC3144296

[B17] Easton N. Marshall F. Fone K. C. Marsden C. A. (2007). Differential effects of the D- and L-isomers of amphetamine on pharmacological MRI BOLD contrast in the rat. Psychopharmacol. Berl. 193 (1), 11–30. 10.1007/s00213-007-0756-5 17387459

[B18] Easton N. Marshall F. H. Marsden C. A. Fone K. C. (2009). Mapping the central effects of methylphenidate in the rat using pharmacological MRI BOLD contrast. Neuropharmacology 57 (7-8), 653–664. 10.1016/j.neuropharm.2009.08.018 19733553

[B19] Farra Y. M. Eden M. J. Coleman J. R. Kulkarni P. Ferris C. F. Oakes J. M. (2020). Acute neuroradiological, behavioral, and physiological effects of nose-only exposure to vaporized cannabis in C57BL/6 mice. Inhal. Toxicol. 32 (5), 200–217. 10.1080/08958378.2020.1767237 32475185

[B20] Ferris C. F. (2022). Applications in awake animal magnetic resonance imaging. Front. Neurosci. 16, 854377. 10.3389/fnins.2022.854377 35450017 PMC9017993

[B21] Ferris C. F. Kulkarni P. Toddes S. Yee J. Kenkel W. Nedelman M. (2014). Studies on the Q175 knock-in model of huntington's disease using functional imaging in awake mice: evidence of olfactory dysfunction. Front. Neurol. 5, 94. 10.3389/fneur.2014.00094 25071696 PMC4074991

[B22] Freels T. G. Baxter-Potter L. N. Lugo J. M. Glodosky N. C. Wright H. R. Baglot S. L. (2020). Vaporized cannabis extracts have reinforcing properties and support conditioned drug-seeking behavior in rats. J. Neurosci. 40, 1897–1908. 10.1523/JNEUROSCI.2416-19.2020 31953372 PMC7046447

[B23] Gardner E. L. (2005). Endocannabinoid signaling system and brain reward: emphasis on dopamine. Pharmacol. Biochem. Behav. 81 (2), 263–284. 10.1016/j.pbb.2005.01.032 15936806

[B24] Han B. Compton W. M. Blanco C. Jones C. M. (2019). Time since first cannabis use and 12-month prevalence of cannabis use disorder among youth and emerging adults in the United States. Addiction 114 (4), 698–707. 10.1111/add.14511 30474910 PMC6411429

[B25] Haney M. Ward A. S. Comer S. D. Foltin R. W. Fischman M. W. (1999). Abstinence symptoms following smoked marijuana in humans. Psychopharmacol. Berl. 141 (4), 395–404. 10.1007/s002130050849 10090647

[B26] Hasbi A. Madras B. K. Bergman J. Kohut S. Lin Z. Withey S. L. (2020). Δ-tetrahydrocannabinol increases dopamine D1-D2 receptor heteromer and elicits phenotypic reprogramming in adult primate striatal neurons. iScience 23 (1), 100794. 10.1016/j.isci.2019.100794 31972514 PMC6971351

[B27] Huestis M. A. Gorelick D. A. Heishman S. J. Preston K. L. Nelson R. A. Moolchan E. T. (2001). Blockade of effects of smoked marijuana by the CB1-selective cannabinoid receptor antagonist SR141716. Archives general psychiatry 58 (4), 322–328. 10.1001/archpsyc.58.4.322 11296091

[B28] Iriah S. C. Trivedi M. Kenkel W. Grant S. E. Moore K. Yee J. R. (2019). Oxycodone exposure: a magnetic resonance imaging study in response to acute and chronic oxycodone treatment in rats. Neuroscience 398, 88–101. 10.1016/j.neuroscience.2018.11.042 30550747

[B29] Jones C. M. McCance-Katz E. F. (2019). Relationship between recency and frequency of youth cannabis use on other substance use. J. Adolesc. Health 64 (3), 411–413. 10.1016/j.jadohealth.2018.09.017 30455035

[B30] Kidorf M. Solazzo S. Yan H. Brooner R. K. (2018). Psychiatric and substance use comorbidity in treatment-seeking injection opioid users referred from syringe exchange. J. Dual Diagn 14 (4), 193–200. 10.1080/15504263.2018.1510148 30332349

[B31] Kilkenny C. Browne W. Cuthill I. C. Emerson M. Altman D. G. NC3Rs Reporting Guidelines Working Group (2010). Animal research: reporting *in vivo* experiments: the ARRIVE guidelines. Br. J. Pharmacol. 160 (7), 1577–1579. 10.1111/j.1476-5381.2010.00872.x 20649561 PMC2936830

[B32] King J. A. Garelick T. S. Brevard M. E. Chen W. Messenger T. L. Duong T. Q. (2005). Procedure for minimizing stress for fMRI studies in conscious rats. J. Neurosci. methods 148 (2), 154–160. 10.1016/j.jneumeth.2005.04.011 15964078 PMC2962951

[B33] Kolb B. Gorny G. Limebeer C. L. Parker L. A. (2006). Chronic treatment with Delta-9-tetrahydrocannabinol alters the structure of neurons in the nucleus accumbens shell and medial prefrontal cortex of rats. Synapse 60 (6), 429–436. 10.1002/syn.20313 16881072

[B34] Koob G. F. Volkow N. D. (2016). Neurobiology of addiction: a neurocircuitry analysis. lancet Psychiatry 3 (8), 760–773. 10.1016/S2215-0366(16)00104-8 27475769 PMC6135092

[B35] LaRue L. Twillman R. K. Dawson E. Whitley P. Frasco M. A. Huskey A. (2019). Rate of fentanyl positivity among urine drug test results positive for cocaine or methamphetamine. JAMA Netw. Open 2 (4), e192851. 10.1001/jamanetworkopen.2019.2851 31026029 PMC6487565

[B36] Lee J. D. Nunes E. V. Jr. Novo P. Bachrach K. Bailey G. L. Bhatt S. (2018). Comparative effectiveness of extended-release naltrexone versus buprenorphine-naloxone for opioid relapse prevention (X:BOT): a multicentre, open-label, randomised controlled trial. Lancet 391 (10118), 309–318. 10.1016/S0140-6736(17)32812-X 29150198 PMC5806119

[B37] Leri F. Bruneau J. Stewart J. (2003). Understanding polydrug use: review of heroin and cocaine co-use. Addiction 98 (1), 7–22. 10.1046/j.1360-0443.2003.00236.x 12492751

[B38] Lewis N. Martinez L. S. (2023). Information scanning impacts nonmedical drug use among college students: a longitudinal study of scanning effects. Health Commun. 38 (10), 2035–2046. 10.1080/10410236.2022.2051269 35332804

[B39] Lupica C. R. Riegel A. C. (2005). Endocannabinoid release from midbrain dopamine neurons: a potential substrate for cannabinoid receptor antagonist treatment of addiction. Neuropharmacology 48 (8), 1105–1116. 10.1016/j.neuropharm.2005.03.016 15878779

[B40] Madularu D. Yee J. R. Kulkarni P. Ferris C. F. (2017). System-specific activity in response to Δ9 -tetrahydrocannabinol: a functional magnetic resonance imaging study in awake male rats. Eur. J. Neurosci. 46 (12), 2893–2900. 10.1111/ejn.13754 29057576

[B41] Maldonado R. (2002). Study of cannabinoid dependence in animals. Pharmacol. and Ther. 95 (2), 153–164. 10.1016/s0163-7258(02)00254-1 12182962

[B42] Manwell L. A. Charchoglyan A. Brewer D. Matthews B. A. Heipel H. Mallet P. E. (2014). A vapourized Δ(9)-tetrahydrocannabinol (Δ(9)-THC) delivery system part I: development and validation of a pulmonary cannabinoid route of exposure for experimental pharmacology studies in rodents. J. Pharmacol. Toxicol. methods 70 (1), 120–127. 10.1016/j.vascn.2014.06.006 24973534

[B43] McMahon L. R. (2011). Chronic Δ⁹-tetrahydrocannabinol treatment in rhesus monkeys: differential tolerance and cross-tolerance among cannabinoids. Br. J. Pharmacol. 162 (5), 1060–1073. 10.1111/j.1476-5381.2010.01116.x 21091643 PMC3051379

[B44] O’Daly O. G. Joyce D. Tracy D. K. Azim A. Stephan K. E. Murray R. M. (2014). Amphetamine sensitization alters reward processing in the human striatum and amygdala. PLoS One 9 (4), e93955. 10.1371/journal.pone.0093955 24717936 PMC3981726

[B45] Panagis G. Vlachou S. Nomikos G. G. (2008). Behavioral pharmacology of cannabinoids with a focus on preclinical models for studying reinforcing and dependence-producing properties. Curr. Drug Abuse Rev. 1 (3), 350–374. 10.2174/1874473710801030350 19630731

[B46] Patton G. C. Coffey C. Carlin J. B. Degenhardt L. Lynskey M. Hall W. (2002). Cannabis use and mental health in young people: cohort study. BMJ 325 (7374), 1195–1198. 10.1136/bmj.325.7374.1195 12446533 PMC135489

[B47] Preece M. A. Sibson N. R. Raley J. M. Blamire A. Styles P. Sharp T. (2007). Region-specific effects of a tyrosine-free amino acid mixture on amphetamine-induced changes in BOLD fMRI signal in the rat brain. Synapse 61 (11), 925–932. 10.1002/syn.20442 17701967

[B48] Ramesh D. Schlosburg J. E. Wiebelhaus J. M. Lichtman A. H. (2011). Marijuana dependence: not just smoke and mirrors. ILAR J. 52 (3), 295–308. 10.1093/ilar.52.3.295 23382144 PMC3606907

[B49] Sadaka A. H. Canuel J. Febo M. Johnson C. T. Bradshaw H. B. Ortiz R. (2023). Effects of inhaled cannabis high in Δ9-THC or CBD on the aging brain: a translational MRI and behavioral study. Front. aging Neurosci. 15, 1055433. 10.3389/fnagi.2023.1055433 36819730 PMC9930474

[B50] Sadaka A. H. Ozuna A. G. Ortiz R. J. Kulkarni P. Johnson C. T. Bradshaw H. B. (2021). Cannabidiol has a unique effect on global brain activity: a pharmacological, functional MRI study in awake mice. J. Transl. Med. 19 (1), 220. 10.1186/s12967-021-02891-6 34030718 PMC8142641

[B51] SAMHSA (2011). Quality CfBHSa. Rockville, MD: HHS.

[B52] SAMHSA (2022). Administration SAaMHS. Rockville, MD: HHS.

[B53] Santos G. C. Marin M. T. Cruz F. C. Delucia R. Planeta C. S. (2009). Amphetamine- and nicotine-induced cross-sensitization in adolescent rats persists until adulthood. Addict. Biol. 14 (3), 270–275. 10.1111/j.1369-1600.2009.00153.x 19523043

[B54] Sathe P. K. Ramdasi G. R. Giammatteo K. Beauzile H. Wang S. Zhang H. (2023). Effects of (-)-MBP, a novel 5-HT(2C) agonist and 5-HT(2A/2B) antagonist/inverse agonist on brain activity: a phMRI study on awake mice. Pharmacol. Res. and Perspect. 11 (5), e01144. 10.1002/prp2.1144 37837184 PMC10576165

[B55] Secades-Villa R. Garcia-Rodriguez O. Jin C. J. Wang S. Blanco C. (2015). Probability and predictors of the cannabis gateway effect: a national study. Int. J. drug policy 26 (2), 135–142. 10.1016/j.drugpo.2014.07.011 25168081 PMC4291295

[B56] Sherrill L. K. Stanis J. J. Gulley J. M. (2013). Age-dependent effects of repeated amphetamine exposure on working memory in rats. Behav. Brain Res. 242, 84–94. 10.1016/j.bbr.2012.12.044 23291159 PMC3566264

[B57] Singh H. Schulze D. R. McMahon L. R. (2011). Tolerance and cross-tolerance to cannabinoids in mice: schedule-controlled responding and hypothermia. Psychopharmacol. Berl. 215 (4), 665–675. 10.1007/s00213-010-2162-7 PMC3140914 21246187

[B58] Slifstein M. van de Giessen E. Van Snellenberg J. Thompson J. L. Narendran R. Gil R. (2015). Deficits in prefrontal cortical and extrastriatal dopamine release in schizophrenia: a positron emission tomographic functional magnetic resonance imaging study. JAMA Psychiatry 72 (4), 316–324. 10.1001/jamapsychiatry.2014.2414 25651194 PMC4768742

[B59] Spencer R. C. Devilbiss D. M. Berridge C. W. (2015). The cognition-enhancing effects of psychostimulants involve direct action in the prefrontal cortex. Biol. Psychiatry 77 (11), 940–950. 10.1016/j.biopsych.2014.09.013 25499957 PMC4377121

[B60] Stamatakis A. M. Stuber G. D. (2012). Activation of lateral habenula inputs to the ventral midbrain promotes behavioral avoidance. Nat. Neurosci. 15 (8), 1105–1107. 10.1038/nn.3145 22729176 PMC3411914

[B61] Stenroos P. Paasonen J. Salo R. A. Jokivarsi K. Shatillo A. Tanila H. (2018). Awake rat brain functional magnetic resonance imaging using standard radio frequency coils and a 3D printed restraint kit. Front. Neurosci. 12, 548. 10.3389/fnins.2018.00548 30177870 PMC6109636

[B62] Stewart J. L. McMahon L. R. (2010). Rimonabant-induced Delta9-tetrahydrocannabinol withdrawal in rhesus monkeys: discriminative stimulus effects and other withdrawal signs. J. Pharmacol. Exp. Ther. 334 (1), 347–356. 10.1124/jpet.110.168435 20375197 PMC2912042

[B63] Tanda G. Goldberg S. R. (2003). Cannabinoids: reward, dependence, and underlying neurochemical mechanisms--a review of recent preclinical data. Psychopharmacol. Berl. 169 (2), 115–134. 10.1007/s00213-003-1485-z 12827346

[B64] Taylor A. Nweke A. Vincent V. Oke M. Kulkarni P. Ferris C. F. (2023). Chronic exposure to inhaled vaporized cannabis high in Δ9-THC alters brain structure in adult female mice. Front. Neurosci. 17, 1139309. 10.3389/fnins.2023.1139309 36950131 PMC10025305

[B65] UNODC (2016). World drug report 2016 vol. Sales No. E.16.XI.7. New York: United Nations Publication.

[B66] Volkow N. D. (2016). Effects of cannabis use on human behavior-reply. JAMA Psychiatry 73 (9), 996. 10.1001/jamapsychiatry.2016.1332 27463420

[B67] Volkow N. D. Baler R. D. Compton W. M. Weiss S. R. (2014b). Adverse health effects of marijuana use. N. Engl. J. Med. 370 (23), 2219–2227. 10.1056/NEJMra1402309 24897085 PMC4827335

[B68] Volkow N. D. Wang G. J. Telang F. Fowler J. S. Alexoff D. Logan J. (2014a). Decreased dopamine brain reactivity in marijuana abusers is associated with negative emotionality and addiction severity. Proc. Natl. Acad. Sci. U. S. A. 111 (30), E3149–E3156. 10.1073/pnas.1411228111 25024177 PMC4121778

[B69] Wilson D. M. Varvel S. A. Harloe J. P. Martin B. R. Lichtman A. H. (2006). SR 141716 (Rimonabant) precipitates withdrawal in marijuana-dependent mice. Pharmacol. Biochem. Behav. 85 (1), 105–113. 10.1016/j.pbb.2006.07.018 16934319

