# Supplementary material for: Chronic exposure to inhaled vaporized cannabis high in Δ9-THC suppresses Adderall-induced brain activity
Source: Front Pharmacol. 2024 Oct 10;15:1413812. doi: 10.3389/fphar.2024.1413812 (PMC11528537; doi:10.3389/fphar.2024.1413812)

Supplementary Figure 1 – Motion Artifact

Supplementary Fig 1 shows the motion artifact as a mean and SE for each of the 250 image acquisitions for all 10 subjects in the two experimental groups – placebo plus Adderall and cannabis plus Adderall. Adderall given to mice exposed to vaporized placebo showed no motion in any orthogonal direction outside 50 um (±) (5.000E-02). The in-plane resolution is ca 187 um^2. These data would indicate that the restraining system and head holder effectively minimize any increase in motor activity caused by Adderall. Note that Adderall given to the placebo group showed no issues with motion artifact. When mice are exposed to vaporized cannabis daily for 10 days and then withheld for 24 hrs – there is increased motion in X and Y due to slight rotation of the head, hence the mirror image of the red and black lines. These spikes between 60-70, 91-101 and plateau starting at 141 reach 100 um (±) (1.000E-01) are just over ½ of a voxel dimension. These changes are judged to be acceptable as most correction algorithms can adjust for motion artifact when movement is below the size of a voxel . The difference between the two experimental conditions is noteworthy because it may reflect withdrawal from chronic cannabis exposure, something not reported in the preclinical imaging literature. We observed a similar increase in motion artifact in another study following a 24 hr hiatus from chronic oxycodone exposure[1]. In that case the motion exceed the dimensions of a voxel and the data were unusable. In the absence of additional data, the possible withdrawal symptoms are purely speculative and will require further research.

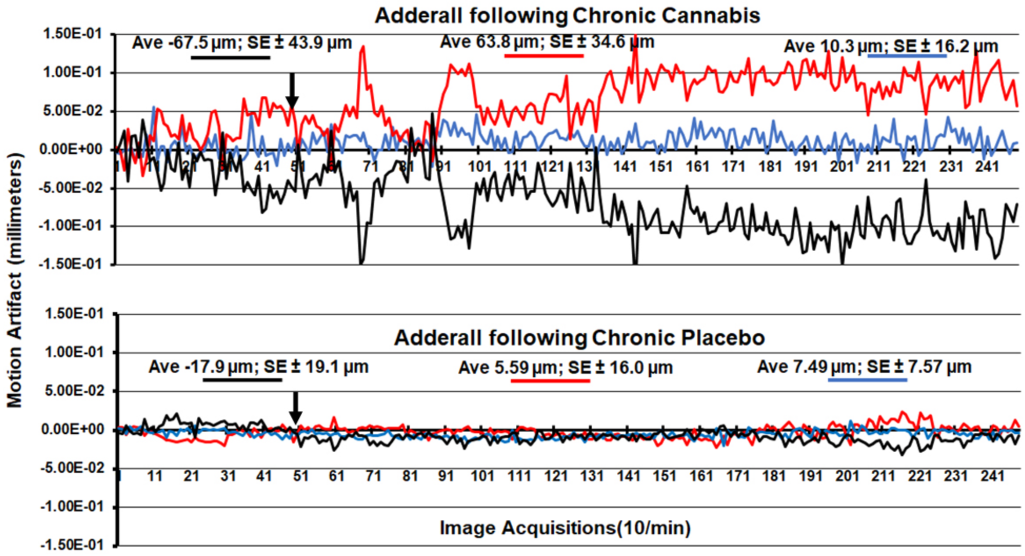

Supplement: Supplementary file 1 [file DataSheet1.zip › Supplementary figure.PDF]
